# Pulsed Electromagnetic Field Affects the Development of Postmenopausal Osteoporotic Women with Vertebral Fractures

**DOI:** 10.1155/2021/4650057

**Published:** 2021-07-16

**Authors:** Wei Liu, Xiao Jin, Zhiqiang Guan, Qiyun Zhou

**Affiliations:** ^1^Department of Orthopedics, Qinghai Provincial People's Hospital, Qinhai, China; ^2^Department of Rheumatology and Immunology, The First People's Hospital of Xuzhou, Xuzhou, Jiangsu 221002, China; ^3^Department of Dermatology, The First People's Hospital of Xuzhou, Xuzhou, Jiangsu 221002, China

## Abstract

**Background:**

Postoperative pain, dysfunction, and significant bone loss may occur after vertebral fractures, which will lead to the occurrence of refractures and shorten the survival time, so postoperative rehabilitation is very important. Pulsed electromagnetic field therapy is noninvasive, pain-relieving, and beneficial to reduce bone loss and is an important treatment for patients to recover after surgery. Therefore, this study analyzed the effect of postmenopausal women's vertebral fracture rehabilitation after pulsed electromagnetic field treatment.

**Method:**

This study uses a randomized controlled study, respectively, in the pulsed electromagnetic field treatment group (40 cases) and the control group (42 cases), respectively. We studied the results of health-related quality of life scores (HRQOL), back pain, body function, hip bone density, bone microstructure of tibia, and radius after 1 month and 3 months after surgery.

**Results:**

Compared with the control group, the pulsed electromagnetic field treatment group (PEMF) can improve significantly the psychological score, 6-minute walk test, and Chair Sit-and-Reach one month after the operation. And at 3 months after surgery, the pulsed electromagnetic field treatment group can improve significantly in health-related quality of life scores (HRQOL), back pain, and body function. Regarding the effect of changes in bone mass, compared with the control group, pulsed electromagnetic field treatment had no significant effect on changes in hip bone density. As a result of changes in bone microstructure, pulsed electromagnetic field treatment can significantly improve the bone microstructure of the radius and tibia three months after vertebral fractures.

**Conclusion:**

Pulsed electromagnetic field therapy has positive significance for improving pain, body functional changes, and bone loss after vertebral fracture surgery.

## 1. Introduction

The risk of refracture within one year after fracture is significantly increased, mainly due to the time from the first fracture and the location of the fracture [1]. So the first fracture is used as a key factor in predicting fractures after surgery. The role of the recency of fracture has been shown for both vertebral and nonvertebral fracture risk. For vertebral fractures, the risk of fractures occurring in the past and then again in the future is also closely related to the number and severity of vertebral fractures. Female patients (≥75-year-old) have a high risk of secondary fractures after fractured vertebrae, with a one- and two-year risk of 14 percent and 26 percent, respectively [2]. At the same time, it is worth noting that the risk of fractures after vertebral fracture surgery is often significantly underestimated, mainly because routine X-ray evaluation often leads to a significant increase in the rate of vertebral fractures [3]. The causes of fractures after fractures are closely related to a variety of factors, including age, sex, changes in intestinal bacteria, and drug factors, the most important of which is bone loss caused by fractures and high morbidity and mortality.

The preventive treatment of refractures after diagnosis of fractures becomes very important [4]. The risk score and vertebral bone density measurement in patients with vertebral fractures are important ways to predict the risk of vertebral fractures at an early stage [5]. Bisphosphonates, selective estrogen-like regulators, parathyroid hormones, and parathyroid hormone-related peptides are all-important treatment methods for preventing vertebral fractures. Other physiotherapy methods, such as electrical stimulation, mechanical stimulation, and hyperbaric oxygen therapy, can significantly improve the speed of fracture healing [6].

Pulsed electromagnetic field (PEMF) therapy began after World War II and is performed by pulsed signals at the damaged site [7], so it is noninvasive. Its main mechanism may be to induce current and treat the target tissue in a noninvasive manner. PEMFs in osteoporosis [8], fractures [9], osteoarthritis, and other aspects have obvious therapeutic effects [10]. The main causes are related to the promotion of osteoblast mineralization and inhibition of osteoclast. At the same time, PEMF also has a good therapeutic effect for pain relief.

In the current study, PEMF can promote fracture regeneration and shorten treatment time in fracture treatment. But PEMF lacked a relevant study for the treatment of osteoporosis after vertebral fracture surgery. The purpose of this study was to analyze the effects of PEMF treatment on fractures and osteoporosis after vertebral fracture surgery.

## 2. Materials and Methods

### 2.1. Participants and Study Design

This study is a randomized controlled study and approved by our Hospital Ethics Committee. Select patients with spinal fractures in our hospitals from January 2018 to June 2020. The patients selected were those aged 60-75 who underwent vertebral fractures and received spinal surgery. All participants who were assigned the same surgery underwent the procedures of percutaneous vertebroplasty as mentioned in previous research [11] in the control group, and the PEMF group. The patients who take part in this study cannot receive treatment for osteoporosis. All enrolled patients signed their informed consent. Vertebral fractures, including clinical vertebral fractures largely captured during unscheduled assessments by the investigator, were identified by a central facility (Synarc, Inc.) using a semiquantitative (SQ) grading scale. A prevalent vertebral fracture was defined as a vertebral body with a semiquantitative grade ≥ 1 at baseline. When compared with the most recent on-treatment spine radiograph, an off-treatment new vertebral fracture was defined by ≥1 grade increase from a previous grade 0 (i.e., normal) in any vertebra between T4 and L4, and an off-treatment worsening vertebral fracture was defined by ≥1 grade increase from a previous vertebral fracture. Both new and worsening vertebral fractures were considered and analyzed as off-treatment vertebral fractures [12]. Multiple vertebral fractures were defined as ≥2 new and/or worsening vertebral fractures confirmed on either a single or serial spine radiograph during the off-treatment period. Nonvertebral fractures required confirmation by a radiologist's report or diagnostic imaging [13]. DXA is also used to measure spinal and hip bone density. The exclusion criteria include diabetes, hypertension, cardiovascular disease, kidney disease, orthopedic diseases other than osteoporosis, and osteoarthritis. The demographic data of the participants are illustrated in Table 1.

### 2.2. Procedure

The study divided the selected vertebral fracture patients into two groups, the control group and the PEMF group, according to the random number table. The above study was carried out following the Helsinki Declaration and with the approval of our Hospital Ethics Committee. We analyzed the clinical results immediately after spinal surgery, 1 month after surgery, and 3 months. Both groups received routine physiotherapy, such as muscle exercises, active or passive activity training, and daily in previous studies [14]. Both groups received calcium (1200 mg) and vitamin D (800 IU) once daily as a basic treatment drug against osteoporosis [15].

PEMF devices were conducted using an XT-2000B therapeutic stimulator (Tianjin xtmed, Tianjin, China). According to the manufacturer's instructions and statement, as well as the purpose of the study, it generated time-varying fields consisting of bursts of asymmetric pulses as in previous studies [16]. According to the manufacturer's instructions and statement, as well as the purpose of the study, it generated time-varying fields consisting of bursts of asymmetric pulses. Each burst lasted for 0.2 ms and was repeated at a frequency of 8 Hz. For the treatment region of the bed, where the lumbar spine of the supine participant is supposed to be, the fields were delivered perpendicular and the flux density within a single burst started with a peak value of 3.82 mT and decreased to 0 mT in 0.2 ms.

### 2.3. Outcome

The main clinical results of this study include health-related quality of life scores (HRQOL), back pain, body function, hip bone density, the bone microstructure of the tibia, and radius.

The health-related quality-of-life score includes two treatment scores, CEOs-16 and EuroQoL. ECOS-16 scores were analyzed in four dimensions: pain, physical function, fear of disease, and psychosocial function [17]. EuroQoL ratings include EQ-5D description ratings and visual simulation ratings [18]. Lumbar back pain assessment is the use of a visual analog scale, which is to indicate the degree of pain in the subjects with 0 to 10.0 is pain-free, and 10 is extremely [19]. In terms of physical function, we used a six-minute walking experiment that required patients to walk a fast distance from the flat, hard ground within six minutes [20]. Chair Sit-and-Reach is to assess the lower body flexibility. This is a safe and socially acceptable test, an alternative to traditional floor sit-and-reach tests in older adults [21].

Radiographic assessment of fusion has been carried by anterior/posterior perspective, and the evaluation of the radiographic fusion was based on three criteria—bony bridging between the two vertebrae, radiolucency at the juncture of the implant and vertebra, and the amount of motion on the dynamic X-rays, as all described in greater detail previously [22]. The assessment of osteoporosis after lumber fracture surgery includes dual-energy X-ray testing (DXA), p-QCT, and bone transformation marker. DXA (Hologic Discovery, Waltham, MA, USA) is to check the lumbar vertebrae density and full hip bone density in preoperative, 3 months, and 6 months after surgery. p-QCT detection is mainly to check the distal radius and tibia. The region of interest was positioned with a 9.5 and 22.5 mm offset from the radius and tibia endplate, respectively, and extended 9.02 mm proximally. Each image comprised 110 slices with an isotropic 82 *μ*m voxel size. The above tests are carried out following the blind law of trained technicians.

### 2.4. Statistical Analyses

This study used the *t*-test and the Fisher test to analyze continuous and classified variables. Use the generalized estimating equation model (GEE) to analyze the average variation between the PEMF group and the control group. We use SAS version 9.3 (SAS 9.3, SAS Institute, Cary, NC) for statistics, and *P* < 0.05 was regarded as statistically significant.

## 3. Results

### 3.1. Characteristics of the Participants

Table 1 shows the demographic characteristics of the participants, and we included 82 patients, including 40 in the PEMF group and 42 in the control group. Both the vertebral fracture group (PEMF group) and the control group underwent PEMF treatment and followed up for 3 months. There were no significant statistical differences between the two groups in terms of sex, weight, and hip bone density.

### 3.2. Effect of PEMF on Spine Function and Quality of Life

To further assess the impact of PEMF on quality of life and function, in Table 2, we first analyzed the pain score of the spine, and the VAS score results showed no significant statistical differences between the two groups at 1 month after surgery. At 3 months after surgery, the PEMF group was significantly better than the control group (*P* = 0.02). For the ECOS-16 score, the PEMF group was also significantly better than the control group in three months after surgery (*P* = 0.01). Besides, for the physical function score, the PEMF group was better than the control group in 1 month (*P* = 0.02) and 3 months (*P* = 0.01) after surgery. For the psychosocial score, the PEMF group was also better than the control group in 1 month (*P* = 0.01) and 3 months (*P* = 0.01) after surgery. For EuroQoL VAS, we found that the PEMF group was also better than the control group in 3 months (*P* = 0.01) after surgery. The result of the six-minute walking experiment also showed that the PEMF group was also better than the control group in 1 month (*P* = 0.03) and 3 months (*P* = 0.01) after surgery. For the Chair Sit-and-Reach right and Chair Sit-and-Reach left, the PEMF group was also better than the control group in 1 month and 3 months after surgery. We also assessed postoperative fusion and found no statistical difference in fusion improvement in the PEMF group compared to the control group 1 month after surgery (*P* = 0.35) but found that the PEMF group was better than the control group 3 months after surgery (*P* = 0.01).

### 3.3. Effect of PEMF on Bone Mass and Microstructure

**Figure 1:**
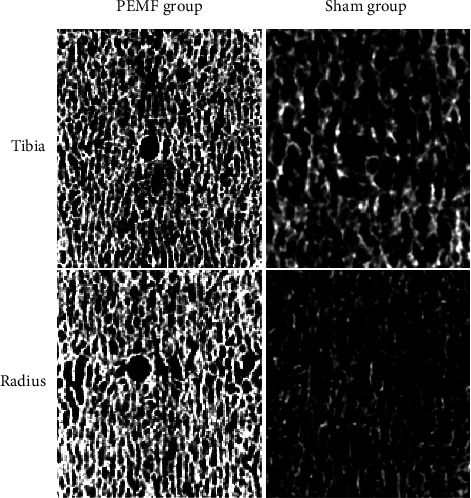
PEMF improves the bone microstructure in the tibia and radius.

To further analyze the effect of a pulsed electromagnetic field on bone mass after spinal surgery, we analyzed the bone mass of the hip and the microstructure of the radius and tibia, respectively. In Table 3, the DXA results found that PEMF showed an increase in hip bone density, but there was no significant statistical difference relative to the control group (Figure 1).

In Table 4, we investigated the influence of bone microstructure, and it was found that for the radius, the PEMF group increased trabecular thickness significantly compared to the control group 1 month after surgery (*P* = 0.04), but there was no significant difference in other indicators. For 3 months after surgery, the PEMF group significantly increased Total vBMD (*P* = 0.02), cortical thickness (*P* = 0.01), BV/TV (*P* = 0.02), trabecular.N (*P* = 0.01), and trabecular thickness (*P* = 0.02). For the tibia microstructure, the PEMF group significantly increased in Total vBMD (*P* = 0.04), BV/TV (*P* = 0.01), and trabecular thickness (*P* = 0.02) in 1 month after surgery. For 3 months after surgery, we found that the PEMF group significantly increased Total vBMD (*P* = 0.02), cortical thickness (*P* = 0.02), BV/TV (*P* = 0.01), trabecular.N (*P* = 0.01), and trabecular thickness (*P* = 0.02).

## 4. Discussion

In this double-blind randomized controlled study, we compared the effects of pulsed electromagnetic waves on postoperative function and bone mass in the treatment of vertebral fractures. It was found that PEMF has only shown significant improvement in physical function score, psychosocial score, 6-MWT, and Chair Sit-and-Reach right within 1 month after surgery. However, PEMF was shown a more significant improvement in patients' quality of life and function in the three months after surgery. The increase in bone density of the hip bone was not significant compared with that of the control group in 1 month and 3 months after surgery, but for the microstructure of the bone, the pulse electromagnetic wave was significantly improved 3 months after surgery.

Osteoporosis and associated osteoporosis fractures are serious public health problems that endanger the health of the elderly. The rate of vertebral fractures in osteoporosis-related fractures is about 15%. The harm of vertebral fracture is manifested in the decrease of the patient's exercise volume and function, which leads to social isolation and depression [23, 24]. The risk of muscle pain after vertebral fracture surgery is very high. The overall rate was 10%-20%, and the female rate was significantly higher than that of male patients [25, 26]. Besides, vertebral fractures are accompanied by a decrease in quality of life and a decrease in life expectancy. Low-energy trauma is an important cause of thoracic and lumbar fractures. Besides, there is a lifetime risk of developing asymptomatic vertebral fracture of 15% [27]. Cooper et al. also found that the five-year survival rate after chest and lumbar fractures is 61%, while the expected survival rate is 76% [28].

For vertebral fracture, treatment methods have pain management, support treatment, and exercise rehabilitation. Postoperative pain management in pain patients includes nonsteroidal anti-inflammatory drugs and painkillers such as qumado. Besides, bisphosphonates also have some improvement in postoperative pain of vertebral fractures [29]. The spine correction brace has obvious effects on stabilizing fractures, preventing deformities, and improving pain symptoms. There is still a lack of relevant research on the treatment of osteoporosis after vertebral fractures. Patients with vertebral fractures are at risk of further fractures after surgery. Active postoperative rehabilitation treatment is an important means of vertebral fracture treatment. Because on the one hand, it can improve the quality of life of patients and also reduce the risk of fractures in the future [30]. Stanghelle et al. found that patients with vertebral fractures in postmenopausal osteoporosis can significantly improve their motor function and quality [31]. Marini et al. found that adapted physical activity exercise can be used to treat patients with chronic diseases. It was found that there was a good therapeutic effect after vertebral fracture surgery [32]. Exercise therapy can increase muscle bone density and prevent falls and fractures [33]. However, whether it is drug therapy or sports rehabilitation therapy, there are potential risks of drug therapy, side effects, and long-term use. Therefore, new methods are needed for vertebral fractures to achieve small trauma and no obvious side effects.

Electrostimulation therapy includes direct current, capacitive coupling, and inductive coupling to promote the spine fusion [34]. Electrical stimulation signals also increase the expression of bone-related genes such as transforming growth factor-*β* superfamily genes (TGF-*β*1, TGF-*β*2, TGF-*β*3, bone morphogenetic protein-2, and morphogenetic protein-4), fibroblast growth factor- (FGF-) 2, osteocalcin (BGP), and alkaline phosphatase (ALP), to promote fracture healing [35], and the effects of this treatment can last up to 12 months [36]. Pulsed electromagnetic field therapy can relieve pain in patients, mainly in a nontoxic and low-risk way to promote the healing and recovery of cell activity [37]. In lumbar vertebral arthritis, pulsed electromagnetic field therapy may also improve neurological symptoms and then improve pain symptoms. Pulse electromagnetic wave therapy also has obvious effects on bone nonconnected treatment. Griffin et al. found that pulsed electromagnetic field therapy has obvious advantages for delayed healing and nonhealing after fractures of long bones [38]. Elshiwi et al. found that adding the pulsed electromagnetic field to conventional physical therapy protocol yields superior clinical improvement in pain, functional disability, and lumbar ROM in patients with nonspecific low back pain than conventional physical therapy alone [39]. Hattapoğlu et al. also found that pulsed electromagnetic wave therapy in cervical disc herniation can be used safely in routine treatment in addition to conventional physical therapy modalities [40]. Pulse electromagnetic wave therapy is well-tolerated, effective with no negative side effects, which can be integrated with rehabilitation for the treatment of chronic and acute pain in musculoskeletal diseases.

Pulsed electromagnetic field therapy also has an obvious effect on osteoporosis treatment. Catalano et al. found that in women with postmenopausal osteoporosis, the evidence of a pulsed electromagnetic wave modulation of RANKL/OPG and Wnt/*β*-catenin signaling pathways was able to explain the metabolic effects of pulsed electromagnetic wave on bone [41]. Parhampour et al. also found that pulsed electromagnetic field therapy can improve bone metabolic disorders and joint function [42]. It has also been found in animal experiments that pulsed electromagnetic field therapy can also improve bone metabolism disorders. Zhou et al. found that pulsed electromagnetic field therapy has a favorable effect on the lumbar spine in this osteoporosis model than did either monotherapy [43]. Jiang et al. found that pulsed electromagnetic field therapy stimulation can prevent bone loss and improve lipid metabolism disorders in glucocorticoid-induced osteoporosis rats. Canonical Wnt signaling pathway plays an important role in bone formation and lipid metabolism during pulsed electromagnetic field stimulation [44]. Elsisi et al. found that PEMFs have better results in bone mineral content and bone mineral density (BMD) in elderly women [45]. After spinal fusion, pulsed electromagnetic wave therapy also showed good results in spinal fusion [46].

Although PEMF has satisfactory therapeutic effects in various musculoskeletal system diseases, PEMF still has some problems. The first is that the mechanism of PEMF is currently unclear and there is no standardized treatment plan and parameters [47]. Secondly, PEMF needs to protect patients and research participants during operation, but whether it poses risks to patients and operators needs further evaluation. Long-term exposure to electromagnetic fields may have adverse effects on the brain and peripheral nervous system, cardiovascular system, cognition, and vestibular function. In addition, electromagnetic field exposure will also lead to an increase in the incidence of depression and other neurodegenerative diseases, so further studies are needed for the adverse reactions caused by PEMF [48].

## 5. Study Limitations

Limitations of this study include the following points. Firstly, the sample size of this study is small, and the lack of long-term follow-up results. At the same time, follow-up time can be up to three months, which leads to the loss of follow-up. At the same time, with the extension of time, the PEMF may have a better effect, but the follow-up time in this study is still relatively short. Secondly, in this study, patients can know whether they are involved in vibration therapy, which may have some long-term effect on patients.

## 6. Conclusion

In our study, we analyzed the effects of pulsed electromagnetic field therapy on vertebral fractures and followed them for 3 months and found that pulsed electromagnetic field therapy showed significant improvements in postoperative pain, quality of life, and function. At the same time, because vertebral fracture surgery is prone to bone loss, so our research further analyzes that pulsed electromagnetic field treatment can further improve bone mass and bone microstructure. This provides new ideas for the future rehabilitation of vertebral fractures.

## Figures and Tables

**Table 1:** Demographic characteristics of the participants.

*N*	PEMF	Control	*P* value
	40	42	
Age, years, mean (SD)	61.5 (2.1)	63.5 (1.2)	0.85
Height, mean (SD), cm	152.61 (1.9)	154.62 (2.8)	0.45
BMI, mean (SD), kg/m^2^	23.5 (2.9)	23.4 (1.4)	0.25
Body weight, mean (SD), kg	61.5 (3.8)	62.3 (4.5)	0.31
Lumbar spine BMD, g/cm^2^, mean ± SD	0.624 (0.0152)	0.694 (0.151)	0.25
Total hip BMD, g/cm^2^, mean ± SD	0.534 (0.021)	0.554 (0.015)	0.21
*T*-score total hip, mean ± SD	&-2.8 (0.4)	&-2.4 (0.1)	0.09
*T*-score lumbar spine, mean ± SD	&-3.8 (0.2)	&-3.9 (0.3)	0.24

PEMF: pulsed electromagnetic field treatment group; BMI: body mass index; BMD: bone mineral density, *P* values derived from mixed-effect models for longitudinal percent change from baseline with a fixed effect for treatment groups, time and adjustments for baseline measurements in an intention-to-treat analysis.

**Table 2:** Effect of pulsed electromagnetic fields (PEMFs) on pain and function.

	Baseline			1 month			3 months		
	PEMF	Control	*P* value	PEMF	Control	*P* value	PEMF	Control	*P* value
Lumbar back pain VAS	7.35 ± 1.25	7.54 ± 0.95	0.51	3.68 ± 0.94	4.02 ± 0.61	0.09	3.05 ± 0.36	3.91 ± 0.48	0.02
ECOS-16	2.51 ± 0.52	2.45 ± 0.25	0.63	2.32 ± 0.35	2.38 ± 0.61	0.25	2.12 ± 0.31	2.42 ± 0.63	0.01
Physical function score	1.84 ± 0.84	1.86 ± 0.25	0.58	1.51 ± 0.62	1.89 ± 0.21	0.02	1.41 ± 0.25	1.86 ± 0.61	0.01
Psychosocial score	2.58 ± 0.25	2.84 ± 0.64	0.65	1.25 ± 0.61	2.42 ± 0.67	0.01	1.36 ± 0.64	2.22 ± 0.12	0.01
EuroQoL VAS	60.31 ± 10.25	63.15 ± 9.25	0.89	73.61 ± 11.62	68.25 ± 12.02	0.59	82.61 ± 13.64	69.36 ± 15.64	0.04
6-MWT	384.61 ± 23.61	381.25 ± 6.58	0.09	415.61 ± 15.26	385.61 ± 15.64	0.03	446.38 ± 23.61	395.15 ± 24.61	0.01
Chair Sit-and-Reach right	90.15 ± 8.61	89.61 ± 3.61	0.58	93.61 ± 4.52	87.61 ± 6.61	0.02	98.64 ± 3.61	88.68 ± 5.93	0.01
Chair Sit-and-Reach left	85.68 ± 5.97	88.94 ± 6.35	0.55	94.81 ± 6.94	89.64 ± 2.98	0.04	96.68 ± 5.21	89.61 ± 5.94	0.02
Radiographic fusion rate	N/A	N/A	N/A	83.6 ± 1.25	80.61 ± 2.61	0.35	86.91 ± 3.1	81.61 ± 5.6	0.01

PEMF: pulsed electromagnetic field treatment group; VAS: visual analog scale/score; ECOS-16: osteoporosis quality of life scoring scale-16; 6-MWT: 6-minute walk test; N/A: not applicable, *P* values derived from mixed-effect models for longitudinal percent change from baseline with a fixed effect for treatment groups, time and adjustments for baseline measurements in an intention-to-treat analysis.

**Table 3:** Effect of PEMF on bone mineral density.

	PEMF	Control	*P* value
Total hip BMD (g/cm^2^)			
1 month	0.692 (0.025)	0.688 (0.089)	0.36
3 months	0.731 (0.042)	0.611 (0.025)	0.06

PEMF: pulsed electromagnetic field treatment group; BMD: bone mineral density, *P* values derived from mixed-effect models for longitudinal percent change from baseline with a fixed effect for treatment groups, time and adjustments for baseline measurements in an intention-to-treat analysis.

**Table 4:** Effect of pulsed electromagnetic fields (PEMF) on bone microstructure.

	Baseline			1 month			3 months		
	PEMF	Control	*P* value	PEMF	Control	*P* value	PEMF	Control	*P* value
Radius									
Total vBMD (g/cm^3^)	193 (3)	198 (1)	0.81	198 (2)	198 (1)	0.25	203 (2)	193 (2)	0.02
Cortical thickness (mm)	0.43 (0.14)	0.41 (0.13)	0.26	0.49 (0.02)	0.43 (0.03)	0.25	0.54 (0.03)	0.40 (0.02)	0.01
BV/TV (%)	7.26 (0.32)	7.35 (0.22)	0.61	7.29 (0.35)	7.01 (0.23)	0.21	7.33 (0.12)	7.00 (0.53)	0.02
Trabecular.N (mm^−1^)	1.21 (0.15)	1.23 (0.11)	0.48	1.35 (0.16)	1.30 (0.21)	0.58	1.45 (0.25)	1.31 (0.35)	0.01
Trabecular thickness (mm)	0.072 (0.0003)	0.075 (0.0018)	0.52	0.073 (0.0014)	0.061 (0.0024)	0.04	0.075 (0.0036)	0.063 (0.0025)	0.02
Tibia									
Total vBMD (g/cm^3^)	181 (3)	180 (1)	0.36	161 (2)	150 (3)	0.04	173 (2)	153 (3)	0.02
Cortical thickness (mm)	0.59 (0.052)	0.58 (0.024)	0.52	0.51 (0.021)	0.50 (0.015)	0.58	0.55 (0.034)	0.48 (0.0024)	0.02
BV/TV (%)	9.36 (0.14)	9.56 (0.85)	0.32	9.84 (0.54)	9.25 (0.94)	0.01	9.88 (0.35)	9.02 (0.69)	0.01
Trabecular.N (mm^−1^)	1.35 (0.03)	1.33 (0.02)	0.48	1.42 (0.01)	1.30 (0.05)	0.25	1.45 (0.05)	1.25 (0.08)	0.01
Trabecular thickness (mm)	0.067 (0.0015)	0.068 (0.0034)	0.28	0.072 (0.0025)	0.061 (0.0025)	0.02	0.075 (0.0021)	0.063 (0.0024)	0.02

PEMF: pulsed electromagnetic field treatment group; BMD: bone mineral density, *P* values derived from mixed-effect models for longitudinal percent change from baseline with a fixed effect for treatment groups, time and adjustments for baseline measurements in an intention-to-treat analysis.

## Data Availability

The data used to support the findings of this study are included within the article.
